# Use of Cytokeratin-19 Concentration to Assess Early Recurrence and Prognosis of Hepatitis B Virus-Related Hepatocellular Carcinoma following Radical Resection in Patients with a Low Serum Alpha-Fetoprotein Concentration

**DOI:** 10.1371/journal.pone.0142727

**Published:** 2015-11-20

**Authors:** Zu-Sen Wang, Wei-Dong Guo, Li-Qun Wu, Xin Yi, Chao Geng, Yu-Jun Li, Ru-Yong Yao

**Affiliations:** 1 Department of Hepatobiliary Surgery, Affiliated Hospital of Medical College Qingdao University, Qingdao, Shandong Province 266003, China; 2 Department of Pathology, Affiliated Hospital of Medical College Qingdao University, Qingdao, Shandong Province 266003, China; 3 Department of Central Laboratory, Affiliated Hospital of Medical College Qingdao University, Qingdao, Shandong Province 266003, China; University Hospital of Essen, GERMANY

## Abstract

Cytokeratin 19 (CK-19) is a prognostic indicator of recurrence and metastasis of hepatocellular carcinoma (HCC) following radical resection. To investigate the role of CK-19 in assessment of early recurrence and prognosis in patients with hepatitis B virus (HBV)-related HCC following radical resection. In total, 235 patients with HBV-related HCC (age, 15–82 years; mean age, 54 ± 10 years) undergoing radical resection were screened for inclusion from January 2005 to December 2010. Malignant tissues and adjacent non-malignant tissues were sampled during surgery, and CK-19 and Ki-67 expression was determined by tissue microarray and immunohistochemistry. CK-19 mRNA levels in 30 randomly selected frozen HCC specimens were examined by reverse transcription polymerase chain reaction from January 2011 to June 2011. Correlations of CK-19 and Ki-67 expression with tumor recurrence, metastasis, disease-free survival (DFS), and overall survival (OS) were analyzed. Elevated CK-19 expression was correlated with early recurrence (*P =* 0.001), shorter DFS (*P =* 0.001), and reduced OS (*P =* 0.010). CK-19 expression was correlated with the Ki-67 index (*P =* 0.037), histological differentiation (*P =* 0.016), focal number (*P =* 0.044), and blood vessel tumor embolism (*P =* 0.002). Patients with metastasis within 1 year exhibited stronger CK-19 expression than did patients without metastasis (*P* < 0.05). Furthermore, early recurrence was associated with elevated CK-19 mRNA levels (χ^2^ = 5.761, *P =* 0.016).When confirmed by a low alpha-fetoprotein concentration (<400 μg/L), CK-19 expression in surgical biopsy specimens taken from patients with HCC during radical resection is an additional valuable indicator of early recurrence, distant metastasis, and poor prognosis in HBV-positive patients.

## Introduction

Hepatocellular carcinoma (HCC) is the sixth most common malignancy worldwide, accounting for 5.7% of all cancers and occurring most commonly in male patients in developing countries [1]. As many as 55% of all cases of newly diagnosed HCC cases occur in China [2]. Furthermore, the incidence of hepatitis B virus (HBV) infection, the predominant cause of HCC, is reportedly as high as 7.18% in China [3]. The high rate of recurrence and metastases following curative resection for HCC remains an important clinical challenge [4]; up to 63% of patients develop recurrence following surgical treatment of HCC [5]. Thus, better prognostic assessment tools for indicating overall survival (OS), recurrence, and distance metastasis in patients with HCC are urgently required.

Cytokeratin 19 (CK-19) is an intermediate filament protein that is critical to epithelial cell structural integrity and used as a highly sensitive biomarker for tumor cell dissemination in the lymph nodes, bone marrow, and peripheral blood [6]. As such, it is a reliable indicator of tumor invasiveness. CK-19 can be used either alone or in combination with other biomarkers to indicate prognosis following curative resection in patients with HCC [7]. An alpha-fetoprotein (AFP) serum concentration of ≥400 μg/L is both a conventional diagnostic factor and prognostic indicator in patients with HCC [8,9]. Furthermore, a low AFP concentration is also an important reference index for the Cancer of the Liver Italian Program (CLIP) score system. Therefore, we use a serum AFP concentration of 400 μg/L as the cutoff value. Moreover, assessment of the CK-19 and AFP concentrations more accurately indicate the prognosis when combined with conventional prognostic indicators for HCC, such as the Ki-67 index [10].

Radical resection is widely considered to be the most effective treatment for HCC in routine clinical practice; however, the curative effect of surgery is often impaired by recurrence or metastasis, which still occur at alarmingly high rates following hepatectomy [11]. Unfortunately, there is controversy regarding the optimal preventative treatments to limit postoperative recurrence in patients with HCC who undergo radical resection, and prolonging disease-free survival (DFS) remains a paramount challenge of HCC research. Therefore, in the present study, we combined prognostic assessment techniques employing CK-19, AFP, and Ki-67 assays to determine HCC prognosis, including recurrence and distant metastasis early after surgery (<1 year). The utility of this combinatorial approach was evaluated in patients infected with HBV, thereby providing preliminary evidence for the importance of more robust prognostic screening of large subpopulations of Chinese patients with HCC.

## Patients and Methods

### Patients

Samples were taken from 235 patients (age, 15–82 years; mean age, 54 ± 10 years) with HBV-positive HCC (HBV-HCC) who underwent radical resection from January 2008 to December 2010 at the affiliated hospital of Medical College Qingdao University. The study protocol was approved by the Ethics Committee of the Qingdao University. Control specimens were taken from adjacent non-cancerous tissue within 2 cm of the incisal margin (81 cases), and intraoperative biopsies were taken from patients with portal hypertension (79 cases). Specimens from 30 patients with HBV-HCC who underwent radical resection from January 2011 to June 2011 were immediately frozen by liquid nitrogen and cryopreserved at −80°C. All patients (or guardians in cases of pediatric patients) signed an informed consent form in accordance with the University policy for medical ethics.

### Inclusion and exclusion criteria

We included patients who (1) were diagnosed with HCC according to the World Health Organization criteria, (2) were positive for HBV at the time of initial diagnosis, (3) were diagnosed with stage I–IV HCC of the TNM Classification of Malignant Tumors (TNM) according to the 7th Edition of the American Joint Committee on Cancer (AJCC) Cancer Staging Manual [12], and (4) had both malignant and adjacent non-cancerous tissues available for sampling during the surgical incision or intraoperative biopsy.

We excluded patients who (1) exhibited any malignancy other than HCC, (2) were not HBV-positive, (3) required preoperative transcatheter arterial chemoembolization, or (4) exhibited any significant contraindications to routine radical resection surgery.

### Sample collection and treatment

During the surgical incision or intraoperative biopsy, samples from malignant and adjacent non-cancerous tissues approximately 2 cm from the tumors were sampled using a direct sampling technique in selected patients. All samples were immediately frozen in liquid nitrogen and cryopreserved at −80°C. Samples of peripheral venous blood were collected during the preoperative period and evaluated using a radioimmunoassay.

### Preparation of samples for microarray

A tissue array instrument (HT-1; France) was used to make 6 × 7 lattice arrays to assess CK-19 and Ki-67 expression. Paraffin blocks of the tissue arrays were cut to 4-μm thickness, stained with hematoxylin and eosin, and observed under optical microscopy (Olympus Bx51; Japan) to confirm the presence of HCC. All tissue array slices then underwent immunohistochemical staining by the two-step PV-6000 method (according to the manufacturer’s instructions). Briefly, phosphate-buffered saline was used to replace the first antibody as a negative control. Rabbit anti-human CK-19 and Ki-67 monoclonal antibodies and immunohistochemical detection reagents were purchased from ZSGB-Bio Company (Beijing, China).

### Immunohistochemical assessments

The slides were randomly allocated for analysis by two independent experienced pathologists with no knowledge of the clinicopathological data. Five high-power fields (×100) containing a minimum of 100 malignant or non-cancerous hepatic cells were examined in each slide, and CK-19-positive cells (CK-19^+^) possessing characteristic brown granules in the cytoplasm were counted and recorded as percentage values. The amounts of CK-19^+^ cells in the tumor and surrounding parenchyma were quantified separately. Specimens were considered CK-19^+^ if ≥5% of the cells were CK-19^+^. The Ki-67 index was the average percentage of Ki-67^+^ cells [13].

### Reverse transcription polymerase chain reaction (RT-PCR)

Total RNA was extracted by using the RNAiso Plus (TaKaRa Biotechnology, Dalian, China) according to the manufacturer’s instructions. Spectrophotometric assay was used to extract the purity of the RNA by a Thermo Scientific NanoDrop 2000c (Thermo Scientific, USA), and cDNAs were reverse-transcribed and amplified by a PrimeScript™ RT-PCR Kit (TaKaRa Biotechnology (Dalian), China) using a PCR Amplifier (Eppendorf Co. Ltd., Hamburg, Germany). Primers were designed using Primer 3 (http://frodo.wi.mit.edu/) and synthesized by the genome sequencing center BGI (Shenzhen, China). Primer sequences for CK-19 were: forward: 5′-ACCAAGTTTGA GACGGAACAG-3′ and reverse: 5′-CCCTCAGCGTACTGATTT CCT-3′. Primer sequences for GAPDH were: forward: 5′-ACCTGACCTGCCGTC TAGAA-3′ and reverse: 5′-TCCACCACC CTGTTGCTGTA-3′. All amplified products of CK-19 were 181 bp. All reactions involved initial denaturation at 95°C for 5 min, followed by 37 cycles at 95°C for 30 s, 58°C for 30 s, 72°C for 35 s, and a final extension of 72°C for 10 min. The PCR products were analyzed by gel electrophoresis in a 2% agarose gel containing ethidium bromide (0.5 mg/ml). Gels were developed by a ultraviolet transilluminator (Vilber Lourmat, France) and photographed. The amplicons were subjected to stripe scanning analysis using an image analysis system, and the levels of CK-19 mRNA were normalized to those of GAPDH.

### Follow-up

After radical resection, all patients were followed monthly for 3 months and at 3-month intervals thereafter until the study end date or death. At each follow-up examination, serum samples were used to assess the AFP concentration, and each patient underwent liver function testing, abdominal ultrasound or computed tomography, and chest computed tomography. Recurrence was diagnosed when imaging examinations revealed unclear areas that were confirmed as recurrence by hepatic arteriography, magnetic resonance imaging, or biopsy. Early recurrence was defined as recurrence within the first year following radical resection surgery. Disease-free survival (DFS) and overall survival (OS) were also recorded. OS was defined as the time from randomization to death of any cause. DFS was defined as the time from randomization to either the first event of recurrent disease or death.

### Statistical analysis

All data were analyzed using SPSS v.13.0 (SPSS Inc., Chicago, IL). Categorical variables were compared by the χ^2^ test and Pearson correlation analysis. Survival was calculated by the Kaplan–Meier method and compared using log-rank tests. Multivariate analyses were performed using a Cox proportional hazards model to identify independent prognostic factors. *P*-values of <0.05 were considered statistically significant (*P* < 0.05).

## Results

### Patient demographics and clinical conditions

Among all 235 HBV-positive patients with HCC who provided samples, the TNM stages were I (*n* = 137), II (*n* = 30), III (*n* = 64), and IV (*n* = 4). Of these, 160 (68.1%) and 75 (31.9%) patients had a preoperative serum AFP concentration of <400 and ≥400 μg/L, respectively. Non-cancerous specimens were taken from the incision site (*n* = 81), and intraoperative biopsy liver specimens were taken from patients with portal hypertension (*n* = 79). Thirty frozen specimens (serum AFP concentration of <400 μg/L) were randomly selected from January 2011 to June 2011. Table 1 details the demographic data and clinical conditions of the study population.

**Table 1:** Demographic and clinical parameters of patients with HBV-related HCC undergoing radical resection.

Parameters	All cases (n)	AFP (ng/ml)	
		<400	≥400
Gender (male/female)	200/35	140/20	60/15
Mean age (range), years	54.22 (15–82)	55.01 (27–82)	52.52 (15–74)
Tumor size (≤ 5/>5 cm)	116/119	89/71	27/48
Edmondson-Steiner classification (I/II/III/IV)	25/134/11/65	23/96/6/35	2/38/5/30
Foci number (1/>1)	204/31	137/23	67/8
Liver capsule invasion (yes/no)	71/164	56/104	15/60
Satellite foci (yes/no)	203/32	146/14	57/18
TNM stage (I/II/III/IV)	137/30/64/4	104/21/33/2	33/9/31/2
Cirrhosis (yes/no)	20/205	12/148	8/57
Vascular tumor thrombosis (yes/no)	208/27	153/7	55/20
Child-Pugh class (A/B)	229/6	158/2	71/4
ALB (>35/≤35g/L)	214/21	146/14	68/7
Tbil (≤22/>22umol/L)	193/42	130/30	63/12
ALT (≤60/>60 U/L)	167/68	113/47	54/21
AST (≤42/>42 U/L)	157/78	111/49	46/29
1-year recurrence (yes/no)	154/81	116/44	38/37

### CK-19 and Ki-67 in HBV-related HCC tissues

**Fig 1:**
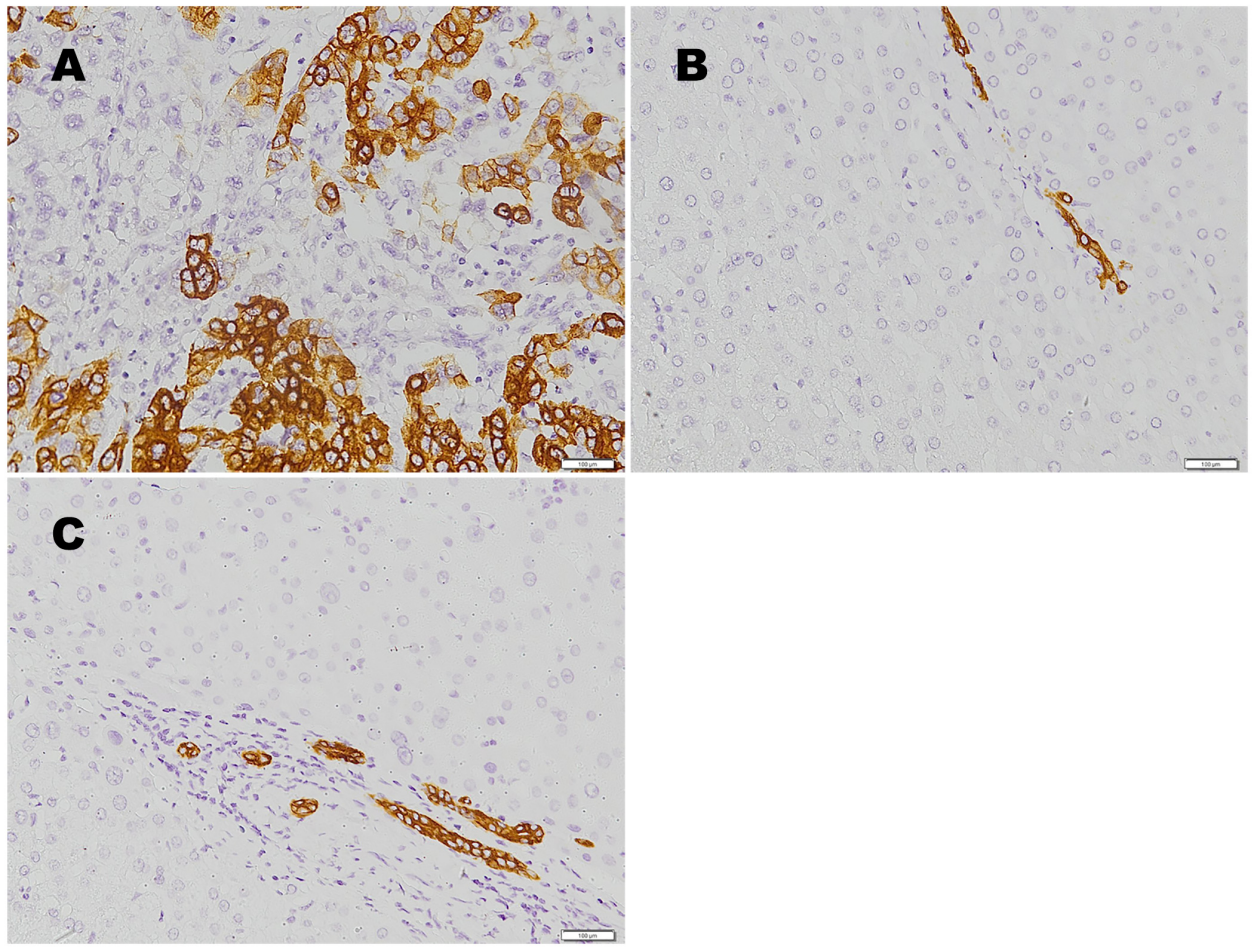
Expression of CK-19 in human primary hepatocellular carcinoma. Immunohistochemical staining of CK-19 in (A) tumor tissues, (B) adjacent non-malignant tissues, and (C) liver cirrhosis tissues. Representative illustrations are shown. Magnification: ×200.

**Fig 2:**
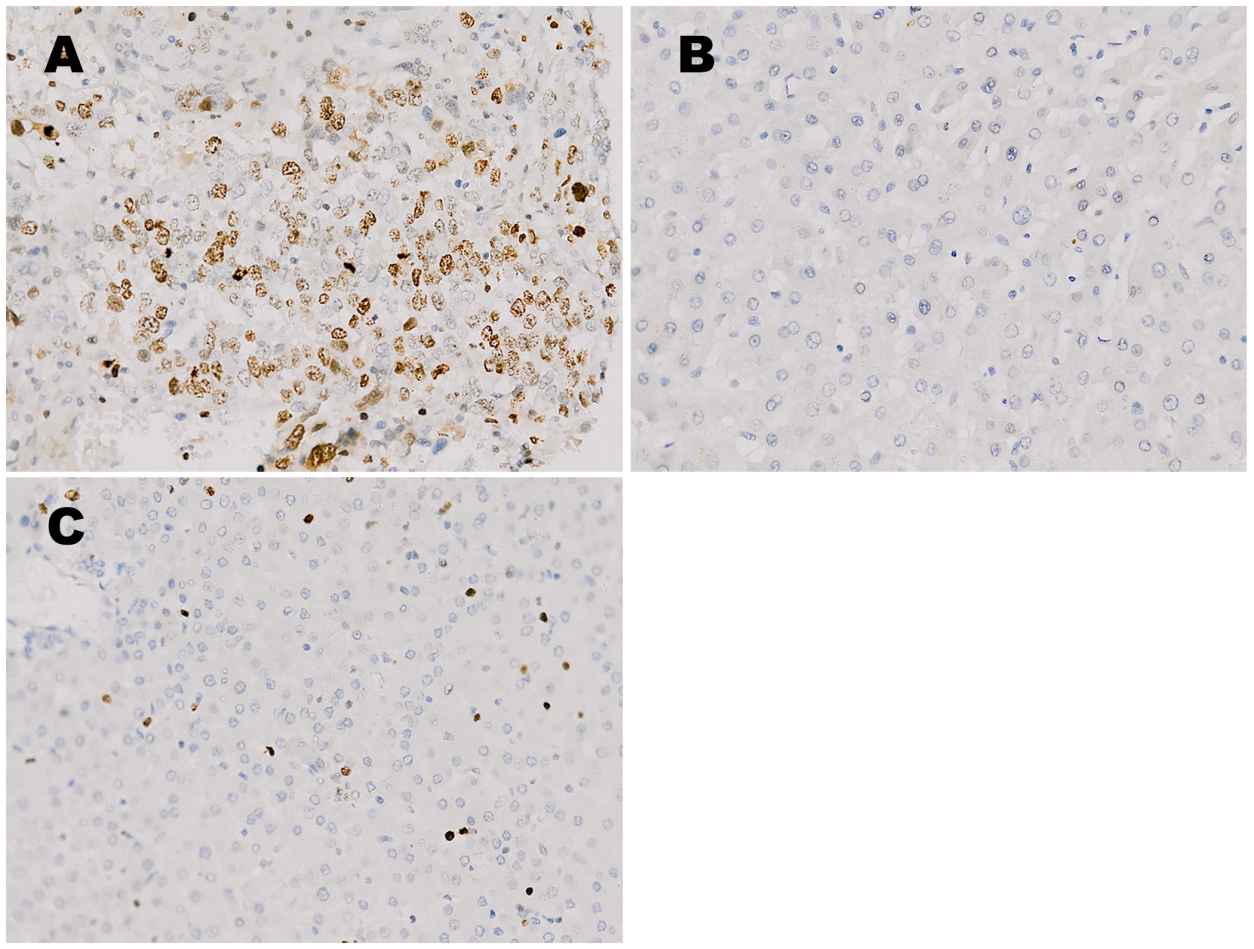
Expression of Ki-67 in human primary hepatocellular carcinoma. Immunohistochemical staining of CK-19 in (A) tumor tissues, (B) adjacent non-malignant tissues, and (C) liver cirrhosis tissues. Representative illustrations are shown. Magnification: ×200.

In the study group, the CK-19^+^ rate was 12.3% (29/235) in HCC tissues and 0.0% (0/235) in adjacent tissues (χ^2^ = 28.8, *P* = 0.000) (Fig 1A–1C). Ki-67 was primarily expressed in the nuclei (Fig 2A–2C). The CK-19^+^ rate in the HCC tissues was 9.4% (15/160) in the AFP < 400 μg/L group and 18.7% (14/75) in the AFP ≥ 400 μg/L group (χ^2^ = 4.08, *P =* 0.044). In the AFP < 400 μg/L group, the Ki-67 index was significantly higher in the CK-19^+^ (23.8%) than in the CK-19^–^ group (17.4%) (ANOVA analysis *P =* 0.037). Relationships between CK-19 expression and clinicopathological factors in patients with an AFP concentration of < 400 μg/L are shown in Table 2. CK-19 expression was significantly related to the degree of histological differentiation (*P =* 0.023), multiple foci (*P =* 0.044), and vascular tumor thrombosis (*P =* 0.002) (Table 2).

**Table 2:** Relationships between CK-19 expression and clinicopathological parameters in patients with an AFP concentration of <400 μg/L (*n* = 160).

		CK-19		χ^2^	*P*-value
		- (%)	+ (%)		
Gender	Male	127 (79.4%)	13 (8.1%)	0.011	0.587
	Female	18 (11.3%)	2 (1.2%)		
Age	>60y	103 (64.4%)	12 (7.5%)	0.541	0.344
	≤60y	42 (26.3%)	3 (1.8%)		
Edmodson-Steiner Classification	I–II	112 (70%)	7 (4.4%)	6.468	0.023
	III–IV	33 (20.6%)	8 (5%)		
Focus	single	127 (79.4%)	10 (6.3%)	4.833	0.044
	multiple	18 (11.3%)	5 (3.0%)		
Tumor Size	>5cm	83 (51.9%)	6 (3.7%)	1.637	0.157
	≤5cm	62 (38.8%)	9 (5.6%)		
Liver Capsule Invasion	No	53 (33.1%)	3 (1.9%)	1.637	0.16
	Yes	92 (57.5%)	12 (7.5%)		
Satellite Focuses	No	134 (83.7%)	12 (7.5%)	2.624	0.129
	Yes	11 (6.9%)	3 (1.9%)		
Cirrhosis	No	9 (5.6%)	3 (1.9%)	3.728	0.088
	Yes	136 (85%)	12 (7.5%)		
Vascular Tumor Thrombosis	No	140 (88.1%)	12 (7.5%)	9.574	0.002
	Yes	4 (2.5%)	3 (1.9%)		
Child-Pugh Class	A	143 (89.4%)	15 (9.4%)	0.21	1
	B	2 (1.3%)	0 (0%)		
TNM Stage	I-II	116 (72.5%)	9 (5.6%)	3.182	0.098
	III-IV	29 (18.1%)	6 (3.8%)		

### CK-19 mRNA is related to recurrence in patients with low AFP concentrations

**Fig 3:**
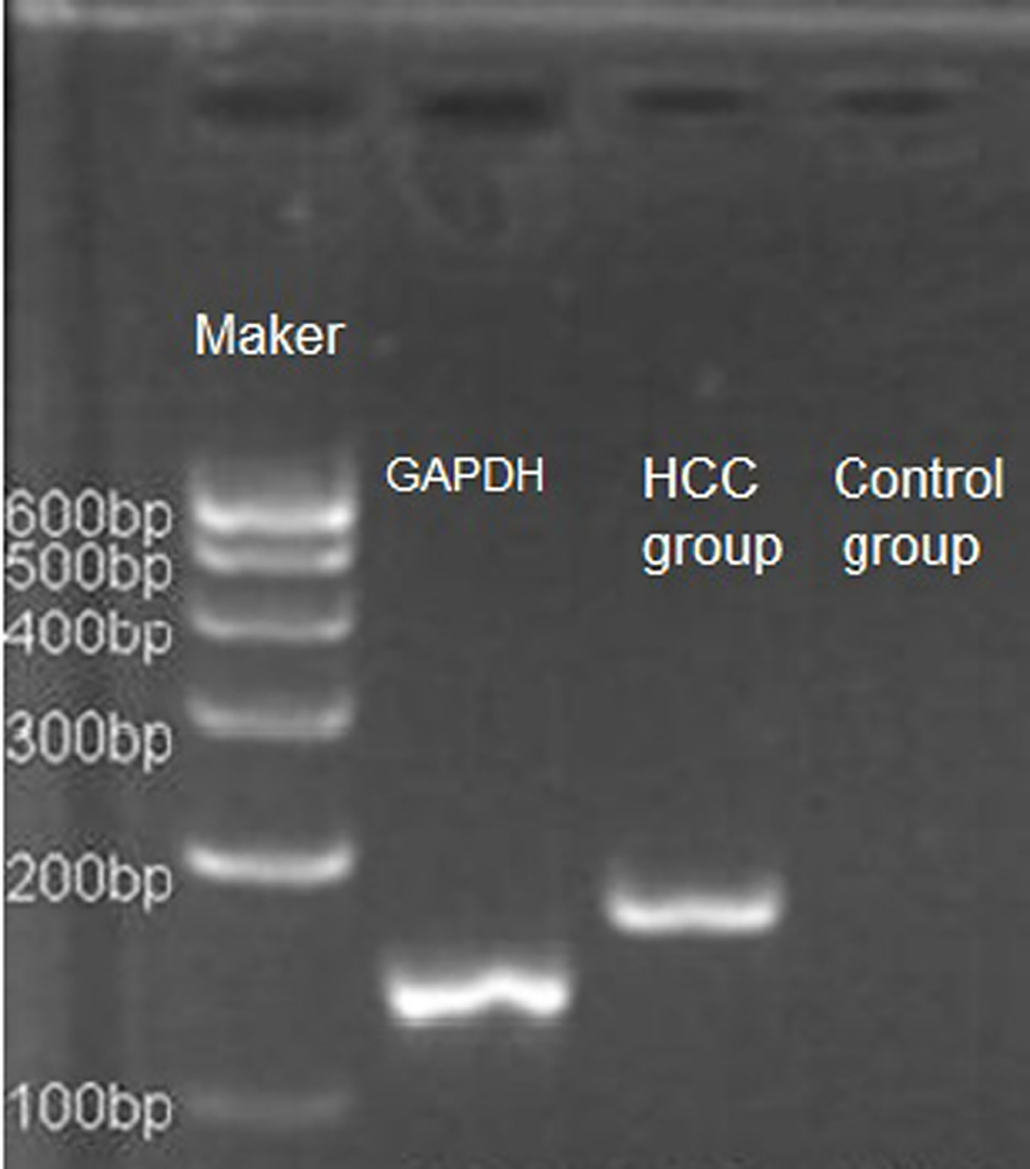
Expression of CK-19 mRNA in HCC tissues (RT-PCR).

The CK-19 mRNA expression in the 30 frozen HBV-infected HCC specimens with low levels of AFP were determined using RT-PCR (Fig 3). The early recurrence rate was 26.7%; the early recurrence rate was higher in the CK-19 mRNA^+^ group than in the CK-19 mRNA^−^group (*P* = 0.002) (Table 3).

**Table 3:** Relationship between CK-19 mRNA and early recurrence (DFS < 12 months) in patients with an AFP concentration of <400 μg/L (*n* = 30).

	n	CK-19 mRNA	F	*P*-value
No recurrence	22	25.80±28.67	10.833	0.002
Early recurrence	8	58.41±30.74		

### CK-19 protein expression in HCC tissue is related to location and rate of metastasis and recurrence

A significant number of patients with extrahepatic metastasis and an AFP concentration of <400 μg/L had CK-19^+^ rates higher than those of patients without extrahepatic metastasis (*P* < 0.05) (Table 4).

**Table 4:** Relationship between CK-19 expression and location of recurrence in patients with an AFP concentration of <400 μg/L (*n* = 160).

	CK-19		χ^2^	*P*-value
	–	+		
No Recurrence	65	4	1.828	0.176
Recurrence	80	11		
No recurrence	65	4	0.004	0.948
Intra-hepatic Recurrence	62	4		
No recurrence	65	4	8.755	0.003
Extra-hepatic metastasis	18	7		
Intra-hepatic Recurrence	62	4	8.213	0.004
Extra-hepatic metastasis	18	**7**		

### Survival analysis

The DFS among patients with AFP concentrations of <400 and ≥400 μg/L was 31.23 (95% CI, 19.29–40.71) and 13.80 (95% CI, 4.51–21.49) months, respectively (log rank *P =* 0.041). The OS was 84.00 and 58.55 months, respectively (Wilcoxon *P* = 0.07). The early recurrence rate was 27.0% (43/160 patients) and 49.3% (37/75), respectively (log rank *P =* 0.001).

No significant differences in the median DFS were observed between CK-19^+^ and CK-19^–^ patients (10 [95% CI, 4.73–15.27] and 25 [95% CI, 16.71–33.28] months, respectively). The OS was significantly lower in CK-19^+^ patients than in CK-19^–^ patients (*P =* 0.023) (Table 5). CK-19^+^ expression in patients with HCC and an AFP concentration of <400 μg/L was significantly related to high early recurrence rates and poor prognosis (Tables 4 and 5).

**Table 5:** Analysis of serum AFP concentration, CK-19 expression, and prognosis in patients with HCC who underwent radical resection.

AFP	CK-19 expression	n	DFS			OS		
			Median (m)	Chi-square	*P-*value	Median (m)	Chi-square	*P-*value
<400 μg/L	+	15	7	6.572	0.010*	19.5	11.816	0.001
	-	145	34			84		
≥400 μg/L	+	61	10	0.038	0.848	53.85	0.076	0.782
	-	14	15			65.33		
Total	+	206	10	3.593	0.058	38	5.188	0.023
	-	29	25			84		

### Implications of the combination of AFP < 400 μg/L and CK-19 expression for early recurrence, metastasis, and overall prognosis in patients with HCC

**Fig 4:**
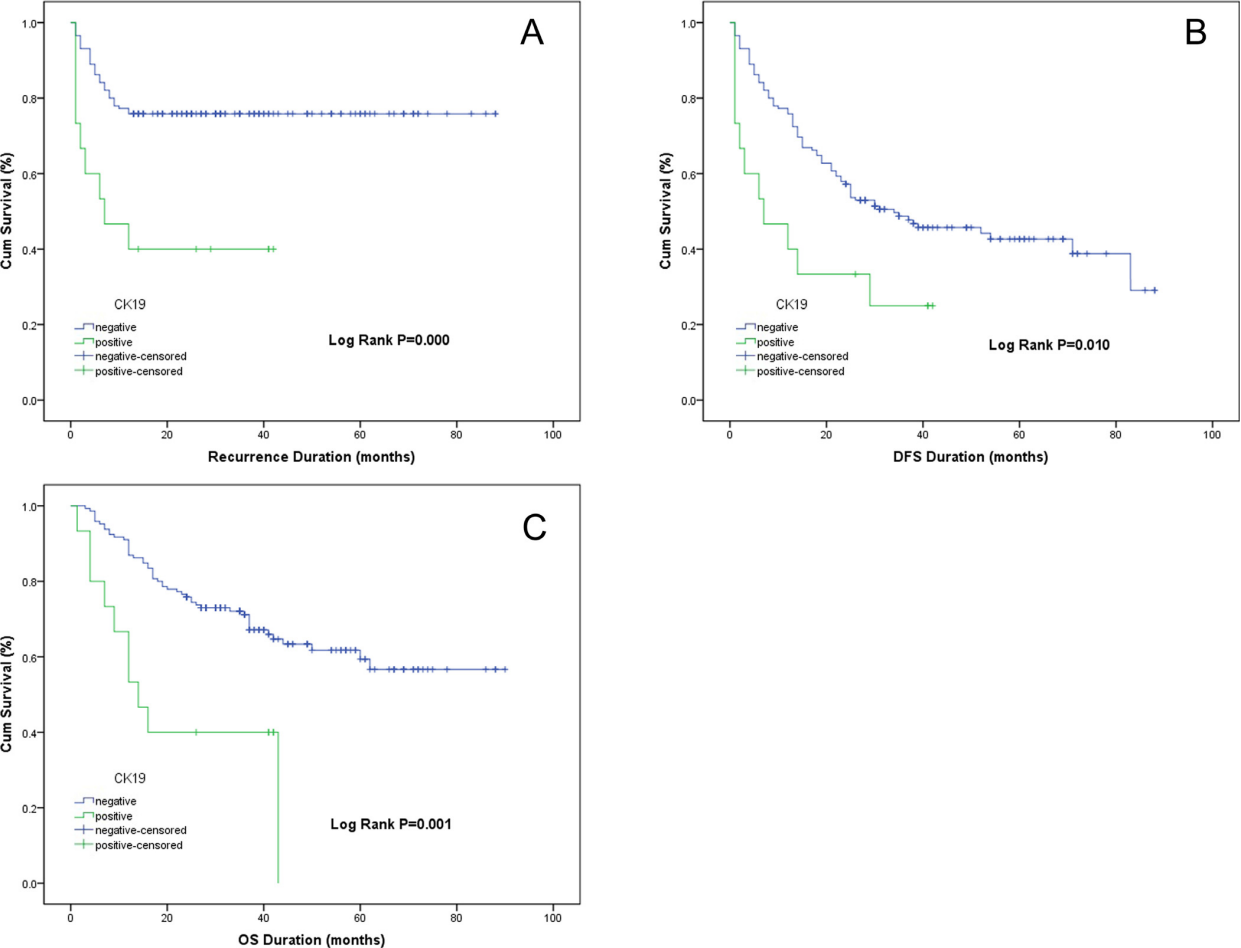
Kaplan–Meier curves based on CK-19 expression level. (A) Early recurrence disease-free survival curves. (B) Disease-free survival curves. (C) Overall survival curves. (A) Early recurrence rate in CK-19^+^ patients was significantly higher than that in CK-19^–^ patients (log-rank test *P =* 0.000). Survival of CK-19^+^ patients was significantly shorter than that of CK-19^–^ patients (DFS: log-rank test *P =* 0.010; OS: log-rank test *P =* 0.001).

Among patients with an AFP concentration of <400 μg/L, the early recurrence rate in CK-19^+^ patients was significantly higher than that in CK-19^–^ patients (53% vs. 23%, respectively; *P =* 0.000) (Fig 4A). The median DFS in CK-19^+^ patients was lower than that of CK-19^–^ patients (11.3 vs. 34.0 months, respectively; log rank *P =* 0.010) (Fig 4B). The median OS of CK-19^+^ patients was significantly lower than that of CK-19^–^ patients (19.5 vs. 84.0 months, respectively; log rank *P =* 0.001) (Fig 4C). Furthermore, Cox regression analysis confirmed that CK-19^+^ expression was an independent risk factor for early recurrence (*P =* 0.043) and OS (*P =* 0.043) (Table 6).

**Table 6:** Cox regression analyses.

		HR	95.0% CI	*P* Value
One-year recurrence	Histological Differentiation	2.175	1.104–4.286	0.025
	CK-19 expression	2.257	4.543–47.514	0.043
	Vascular tumor thrombosis	4.97	1.726–14.306	0.003
Overall survival	Histological differentiation	2.047	1.130–3.708	0.018
	Vascular tumor thrombosis	3.496	1.170–10.447	0.025
	CK-19 expression	2.208	1.024–4.759	0.043

## Discussion

The current study indicated that combined CK-19 and Ki-67 assessment can be used to indicate the risk for poor prognosis, including recurrence and distant metastasis in the first year following radical resection for HCC in HBV-positive patients, particularly in patients with an AFP concentration of <400 μg/L. This combinatorial strategy may provide a useful basis for risk assessment and early implementation of preventative strategies that can aid in improving DFS and OS in Chinese patients with HBV-related HCC.

Previous studies have shown that patients with HCC and a serum AFP concentration of ≥400 μg/L have significantly shorter median survival times than do patients with lower serum AFP concentrations [14]. Thus, a serum AFP concentration of 400 μg/L was applied as a boundary point in the current study and revealed that patients with a lower AFP concentration and CK-19^+^ tumor tissues were more likely to experience a poor prognosis, including both early recurrence and distant metastasis. Wu *et al*. [15] reported that distinct HCC cell lines variably upregulate CK-19, which may be related to the invasiveness of specific HCC tumors as determined mechanistically by the amount of CYFRA 21–1 generated by these cells.

Notably, the current study demonstrated no progressive relationship between CK-19^+^ expression and postoperative recurrence or prognosis in patients with an AFP concentration of ≥400 μg/L that would indicate a relationship with tumor development or stage, suggesting that CK-19^+^ may indeed be an important predictor of early recurrence and prognosis for patients with an AFP concentration of <400 μg/L that is specific to certain HCC cell lines. However, a further mechanistic study will be required to fully determine the pathways involved in CK-19 upregulation and potential strategies for mediating these effects.

Several studies have reported widely variant relationships between CK-19 expression and HCC prognosis, with the result that potentially useful clinical assays have not been widely implemented using CK-19. Xiang *et al*. [16] and Uenishi *et al*. [17] both reported that CK-19 upregulation was related to a reduced DFS time, consistent with the present study. Conversely, Yang *et al*. [5]reported that CK-19 did not clearly indicate a poor prognosis or recurrence when applied as an isolated assessment. Thus, combinatorial methods may aid in improving the reliability of HCC prognostic assessments when coupled with low AFP concentrations and consistent Ki-67 index results, which reflect tumor proliferation [18]. This is consistent with the findings of Maeda *et al*. [18], who found that Ki-67 combined with clinicopathological features could indicate the prognosis of HCC with relatively good accuracy. Thus, further development and evaluation of clinical strategies that combine both conventional and novel prognostic indicators, such as CK-19, are critical to improving prognostic assessment and survival in patients with HCC.

The relationship between CK-19 upregulation and metastasis was underscored by the findings of the current study, which demonstrated that patients with CK-19^+^ HCC were more likely to exhibit active tumor proliferation and extra-hepatic metastasis. These results are consistent with recent findings that upregulation of CK-19 was associated with metastatic progression in breast cancer cell lines [19]. This mechanism, however, remains debatable. Alix-Panabières *et al*. [19] suggested that CK-19 may be related to protein blebbing of the cytoplasm of tumor cells, analogous to viral budding, or alternatively to the release of exosomes containing cytoskeletal proteins. Based on the current findings, however, CK-19, which is expressed in the cytoplasm and cytomembrane of HCC cells, may be directly secreted into the extracellular matrix, where is hypothetically involved in initiation of a signaling cascade involving cytomembrane integrin, thereby inducing various matrix-degrading enzymes such as MMP-9 [20, 21]. Furthermore, CK-19 and CK-14 also reportedly induce self-antibodies, potentially leading to immunoreactions that promote transference and migration of tumor cells [22]. Although these hypotheses suggest that the complete mechanism of the variable association between CK-19 with poor prognosis will require further biochemical studies for confirmation, the current study indicates that CK-19 applied in combination with conventional prognostic assessment markers may be superior for indication of recurrence and metastasis in patients with HCC.

The current findings provide a comprehensive overview of a controversial clinical issue for HCC prognostic assessment; however, they are not without their limitations. The relatively small sample size and inclusion of only Chinese patients may mean that these results are not widely applicable to other populations. Furthermore, the relatively small sample size may limit the power of statistical analysis, particularly for characteristics that are underrepresented in the current cohort. Thus, further biomechanical and clinical studies will be required to confirm these results. However, based on these findings, we recommend the addition of CK-19 measurement to the clinical prognostic tools for HCC in conjunction with other conventional instruments, such as Ki-67 analysis.

When coupled with a low serum AFP concentration (<400 μg/L) and a consistent Ki-67 index, upregulation of CK-19 can be a powerful additional prognostic indicator for use in patients with HBV-related HCC undergoing radical resection. It can be inferred from these results and previous publications that HCC cells with characteristically high levels of CK-19 are more invasive and thus more likely to cause early recurrence and metastasis in affected patients. In addition to routine clinical prognostic assessments, CK-19 expression should be analyzed in biopsy samples from patients undergoing radical resection, thereby providing an additional valuable marker for the risk of potential recurrence and metastasis. Further studies of HCC cell lines with upregulated CK-19 expression will be required to assess the mechanisms of tumor progression and potential therapeutic options for treating patients with HCC.
